# Integrating GWAS, linkage mapping and gene expression analyses reveal the genetic control of first branch height in *Brassica napus* L

**DOI:** 10.3389/fpls.2022.1080999

**Published:** 2022-12-15

**Authors:** Zhixue Dong, Minqiang Tang, Xiaobo Cui, Chuanji Zhao, Chaobo Tong, Yueying Liu, Yang Xiang, Zaiyun Li, Junyan Huang, Xiaohui Cheng, Shengyi Liu

**Affiliations:** ^1^ National Key Lab of Crop Genetic Improvement, College of Plant Science and Technology, Huazhong Agricultural University, Wuhan, China; ^2^ Key Laboratory of Biology and Genetic Improvement of Oil Crops, the Ministry of Agriculture and Rural Affairs of the People's Republic of China (PRC), Oil Crops Research Institute, Chinese Academy of Agricultural Sciences, Wuhan, China; ^3^ Key Laboratory of Genetics and Germplasm Innovation of Tropical Special Forest Trees and Ornamental Plants, Ministry of Education, School of Forestry, Hainan University, Haikou, China; ^4^ Guizhou Rapeseed Institute, Guizhou Academy of Agricultural Science, Guiyang, China

**Keywords:** *Brassica napus*, first branch height, RIL, GWAS, *TCP1*

## Abstract

Rapeseed (*Brassica napus* L.) is a crucial oil crop cultivated worldwide. First branch height, an essential component of rapeseed plant architecture, has an important effect on yield and mechanized harvesting; however, the underlying genetic mechanism remains unclear. In this study, based on the 60K single nucleotide polymorphism array and a recombinant inbred lines population derived from M083 and 888-5, a total of 19 QTLs were detected in five environments, distributed on linkage groups A02, A09, A10, C06, and C07, which explained phenotypic variation ranging from 4.87 to 29.87%. Furthermore, 26 significant SNPs were discovered on Chr.A02 by genome-wide association study in a diversity panel of 324 re-sequencing accessions. The major QTL of the first branch height trait was co-located on Chr.A02 by integrating linkage mapping and association mapping. Eleven candidate genes were screened *via* allelic variation analysis, inter-subgenomic synteny analysis, and differential expression of genes in parental shoot apical meristem tissues. Among these genes, *BnaA02g13010D*, which encodes a TCP transcription factor, was confirmed as the target gene according to gene function annotation, haplotype analysis, and full-length gene sequencing, which revealed that TATA insertion/deletion in the promoter region was closely linked to significantly phenotypic differences *BnaA02.TCP1* ^M083^ overexpression resulted in decreased branch height and increased branch number in *Arabidopsis*. These results provide a genetic basis for first branch height and the ideal architecture of *B. napus*.

## Introduction

Rapeseed (*Brassica napus* L.) is an important source of edible oil, biodiesel and protein feed for livestock in the world (Chalhoub et al., 2014). It is the third largest oil crop with an annual seed yield of 72 million tons that takes up ~13% of global oilseed production after oil palm and soybean (FAOSTAT, https://www.fao.org/faostat/). Global rapeseed production has increased significantly since 1961, but there has been no change in seed yield, at least in the last five years (Liu et al., 2022). With the growing human population and changing environment, the energy demand for rapeseed and crop production has increased. However, owing to the increasing urbanization, dwindling resources and competition with staple crops for land, the development of the rapeseed industry has plateaued in recent years (Liu et al., 2022). Therefore, there is an urgent to study and cultivate high-yield rapeseed varieties using genomics and biotechnology to meet global demands.

Crop yield mainly comprises the number of plants per unit area, number of grains per plant, and thousand-seed weight. Plant architecture, defined as the three-dimensional organization of the plant body, plays an extremely important role in crop growth, yield, and harvest index (Reinhardt and Kuhlemeier, 2002; Sarlikioti et al., 2011; Gou et al., 2017; Wang et al., 2017; Sun et al., 2019; Tian et al., 2019; Yang et al., 2019; Zheng et al., 2020; Guo et al., 2020). Crop breeding research has focused on shoot architecture, including the component traits of plant height, branch or tiller number and angle, leaf shape, size and angle, and inflorescence morphology (Wang and Li, 2008; Wang et al., 2018). Among these traits, plant height is the most prominent determinant of plant architecture and is often selected in domesticated crops. In the 1960s, the central theme of the “Green Revolution” was reducing plant height by introducing the semi-dwarf gene *sd1* into rice and the dwarf genes *Rht-B1b*/*Rht-D1b* into wheat, which greatly improved lodging resistance and the harvest index (Khush, 2001; Liu et al., 2018). The upright and sparsely branched phenotype of maize (*Zea mays*) is suitable for high-density planting, which greatly improved the harvest index (Tian et al., 2019). Currently, one of the major challenges in modern agriculture is the development of an ideotype that produces high yields in various environments.

Plant architecture is controlled by a complex regulatory network that includes phytohormones, miRNAs, and several transcription factors (Lo et al., 2017; Tang and Chu, 2017; Liu et al., 2019; Schneider et al., 2019; Chen et al., 2020; Guo et al., 2020). The molecular mechanisms regulating plant aerial architecture have been identified in several crops, including rice (*OsIPA1*), maize (*ZmUPA2*, *Zmtin1*), wheat (*TaSPL8*), cucumber (*CsBRC1*), soybean (*GmWRI1b*), and rapeseed (*BnaMAX1*) (Liu et al., 2019; Shen et al., 2019; Sun et al., 2019; Tian et al., 2019; Zhang et al., 2019c; Zheng et al., 2020; Guo et al., 2020), which improved plant performance and yield. Simultaneously, most identified genes that shape plant architecture are related to plant height, which participates in biosynthesis or signaling pathways of phytohormones, including auxin, gibberellins, brassinosteroids and strigolactones (Wang et al., 2018). Genetic studies in tomato, *Arabidopsis*, rice, and maize have shown that many genes are directly involved in the initiation and outgrowth of axillary buds, which significantly affects crop yield by influencing branch or tiller numbers (Wang and Li, 2006; Wang et al., 2016; Wang and Jiao, 2018).

Dicot plant architecture is typical of rapeseed plants, including the shoot apical meristem (SAM), which establishes the shoot as the primary growth axis of the plant by continuously initiating phytomers, and the root apical meristem, which establishes the primary root that can branch to form secondary or higher-order lateral roots (Teichmann and Muhr, 2015). Recently, an ideotype-like rapeseed plant stature mutant with compact inflorescence and high pod density was isolated, which could be utilized to shorten the flowering period and increase production and was highly desired for mechanized harvest (Fan et al., 2021; Kuai et al., 2022). In general, rapeseed with semi-dwarf plant type tends to show many branches and high primary branch height, which is beneficial for improving production by increasing pod number per plant and enhancing lodging resistance. Therefore, first branch height (FBH), the distance between the growing point of the first effective branch and the cotyledon node, is also a non-negligible factor shaping rapeseed plant architecture and influencing rapeseed yield (Zheng et al., 2019). Nevertheless, there are no reports on FBH gene localization and cloning in rapeseed.

Quantitative trait loci (QTL) mapping combined with genome-wide association study (GWAS) is an effective approach for identifying loci controlling complex traits (Han et al., 2018; Wang et al., 2020b). Zhao et al. (2016) detected 27 QTLs related to FBH trait distributed on 11 chromosomes with a doubled haploid population derived from KenC-8 and N53-2. Cai et al. (2016) also used a doubled haploid population derived from Huashuang 5 and J7005 to detect16 QTLs that contribute to FBH trait in rapeseed. Luo et al. (2017) genotyped and reanalyzed the previous phenotypes of a doubled haploid population (Tapidor and Ningyou7), and detected 35 QTLs related to FBH, which were distributed on A01, A02, A03, A05, A06, A09, A10, C05, C06, C07, C08, and C09. The contribution rate of these QTLs ranged from 3.19 to 22.91%. Shen et al. (2018) detected five plant height-related QTLs and five FBH-related QTLs on chromosomes A02 and A07 using a doubled haploid population derived from Westar and Y689. All the above reports identified QTLs for rapeseed FBH, but all QTLs were screened using a single method. QTL mapping and GWAS can accurately and rapidly locate and screen for candidate genes. Recently, several studies detected and screened candidate genes for agronomic traits in rapeseed (Sun et al., 2016; Wang et al., 2020b; Zhao et al., 2022). However, this approach was not used to rapeseed FBH.

In this study, a recombinant inbred lines (RILs) population derived from M083 and 888-5 containing 210 lines was used for linkage mapping to detect QTLs for FBH in different environments with a 60 K Brassica Infinium single nucleotide polymorphism (SNP) array. We further identified several candidate genes for FBH with a GWAS of 324 natural accessions that our lab collected and re-sequenced previously based on QTL mapping. The results obtained here lay the foundation for further study of the genetic architecture and molecular mechanisms of FBH in rapeseed, and facilitate molecular breeding to improve plant architecture for easy mechanical harvest and increase rapeseed yield.

## Materials and methods

### Plant materials

An association panel composed of 324 diverse rapeseed inbred lines (25 winter, 259 semi-winter, and 40 spring types) collected worldwide was used for GWAS. These lines were grown in Yangluo, Wuhan, China in 2016, 2017, 2018, and 2019. Each variety was planted in a plot with three rows (33-cm line width and 20-cm plant distance), with 12 plants per row. The lines were randomly designed with three replicates.

A RIL population containing 210 lines derived from the crossing of 888-5 and M083 was used in a previous study. The parent line 888-5 had a lower FBH than the M083 inbred line (Liu et al., 2005). The genetic linkage map contained 9,278 SNPs covering 4,071.08 cM, which mapped to 2,771 loci (bins) in a previous study (Zhang et al., 2019b). The population was planted in the standard breeding trial fields of Xiantao and Wuhan, Hubei Province, and Yangzhou, Jiangsu Province, China in 2016 and 2017. The planting method was the same as that of association population.

### Phenotyping and data analysis

At maturity, the association population, RIL population, and the parental lines (888-5 and M083) were assessed for FBH (from the base of the stem to the first effective branch) in each environment. The phenotypic values of the lines in each environment were calculated as the average of ten plants in triplicate. The two parents were compared using two-sample *t*-test. SPSS 21 (Armonk, NY, United States) was used to calculate the frequency distribution. Analysis of variance was performed using the GLM procedure of SAS. The best linear unbiased prediction (BLUP) of each inbred line in the association population and the effects of genotype, environment, and genotype × environment interactions in linkage and association mapping population were obtained by using the “*lmer*” function in the “*lme4*” package of R version 3.6.3 (http://www.R-project.org). The broad-sense heritability (*H* ^2^) of FBH was calculated as follows:


$$H^{2}=G/(G+\frac{G\times E}{n}+\frac{e}{nr})$$

where G is the genetic variance, G×E is the variance of the genotype-environment interaction, e is the residual error variance, n is the number of environments, and r is the number of replicates within the environment.

### QTL identification and analysis

The QTL for FBH was detected using composite interval mapping(CIM) (Zeng, 1994) in Windows QTL Cartographer 2.5 (Wang et al., 2005). The QTL evidence was checked at 1cM intervals with a 10 cM window size using the likelihood ratio test (LRT), after which the LRT values were converted to a logarithm of odds (LOD) scale (LOD¼; 0.217_LRT). The threshold LOD value was estimated using 1,000 permutations at a significance level of *P*< 0.05. The confidence interval of the QTL position was determined by the 2-LOD interval method. The contribution rate (R^2^) and additive effect of a presumptive QTL were simultaneously calculated using Windows QTL Cartographer 2.5. The QTL nomenclature described by McCouch et al. (1997) was adopted in this study, each designation was named begins with ‘*q*’ followed by the abbreviation of the trait name, the linkage group, and the serial number of the QTL on the linkage group, such as *qFBH*02-1. The QTLs position was drawn using MapChart software (Voorrips, 2002).

### GWAS for FBH

GWAS for FBH was performed using a mixed linear model (MLM) to detect associations between phenotypes and genotypes in TASSEL v5.0 (http://www.maizegenetics.net/tassel) (Zhang et al., 2010). The population structure and genetic relationships between accessions were also calculated using TASSEL v5.0 in previous study (Zhang et al., 2019b). The *P* value was calculated for each SNP as *P* = 1/N according to the adjusted Bonferroni method, where N is the number of SNPs calculated by the simple program in R software (Li et al., 2012; Li et al., 2021), and defined as the genome-wide control threshold. The quantile-quantile (Q-Q) plot shown with the expected *P*-value and Manhattan plot were displayed using the R package “*qqman*” (Turner, 2014). Linkage disequilibrium (LD) of the whole genome was analyzed using PopLDdecay software (Zhang et al., 2019a). The LD heat map of 0.5 LD sequence regions adjacent to the significantly associated SNPs were generated using the “*LD heatmap*” package in R.

### RNA extraction and gene expression quantification

Parental SAM samples were collected at the initial developmental stage of the shoot bud. Total high-quality RNA was extracted using the FastPure Plant Kotal RNA Isolation Kit (Vazyme, Nanjing, China). Then, cDNA was synthesized using HiScript III 1st Strand cDNA Synthesis Kit (Vazyme, Nanjing, China). Quantitative real-time PCR (qRT-PCR) was performed using SYBR Green Real-time PCR (Takara, Kusatsu, Japan) in a CFX Connect Real-time PCR system (BioRad, United States), and *BnaActin* was used as the reference gene. The 2^-∆∆CT^ method was used to calculate the relative gene expression levels (Livak and Schmittgen, 2001). Each qRT-PCR experiment contained three technical replicates. The specific primers used for qRT-PCR are listed in **Supplementary Table 1**. Genes were considered as differentially expressed between parents when the log_2_fold change was ≥ 1 or ≤ -1, and they can be selected as candidate genes.

### Gene analysis in the common interval

Allele variation and sub-genome synteny analysis were used to exclude genes when predicting candidate genes for FBH (Zhang et al., 2019b). Based on the *B.napus* cv. Darmor-*bzh* reference genome (Chalhoub et al., 2014), we matched the genetic markers for linkage mapping to the physical positions, and compared the linkage and association mapping results. Then, parallel region was defined as co-located interval, which is the major QTL for trait. Parental allele variation analysis was performed using homozygous polymorphic SNP and InDel derived from deeply re-sequenced parent data. The genes within the major QTL were analyzed using sub-genome synteny, and synteny genes existed in another sub-genome of *B.napus* were excluded. Candidate genes were identified combined with gene expression levels derived from qRT-PCR data.

### Haplotype analysis of candidate genes

The 324 accessions of the association population were used to analyze the haplotypes of candidate genes in the genomic regions. The SNPs within the 1.5 kb promoter region of the candidate genes and the nonsynonymous SNPs in the coding sequence were used for haplotype analysis.

### Analyses of parental *BnaA02.TCP* sequence

The total parent DNA was extracted from the leaf samples using the Hi-DNA secure Plant Kit (TianGen, Beijing, China). The sequence of the genomic regions ~1.5 kb upstream of the *BnaA02.TCP1* coding sequence was cloned using the primers listed in **Supplementary Table 2**. The cloned products were sent to QingKe Biotechnology Co., LTD for sequencing, and the genomic sequences were compared using MEGA-X (Tamura et al., 2011).

### Construction of the *35S::BnaA02.TCP1* ^M083^molecular cassette and plant transformation

The *BnaA02.TCP1* ^M083^ coding sequence was isolated using the primers listed in **Supplementary Table 2** to generate the *35S::BnaA02.TCP1* ^M083^ construct. The PCR fragment was cloned into the pBI121 vector and was driven by the 35S promoter. *Agrobacterium tumefaciens* strain GV3101 carrying the sequenced construct was confirmed *via* colony PCR and transformed into col-0 plants using the floral dipping method in *Arabidopsis*. Surviving seedlings with kanamycin were positive transgenic plants and were validated using qRT-PCR experiments, *AtActin* was used as the reference gene and three repeats of samples were collected for RNA extraction. Six transgenic lines were obtained, three of which were randomly chosen for phenotypic observation.

## Results

### Phenotypic variation in RIL and association populations

**Figure 1:**
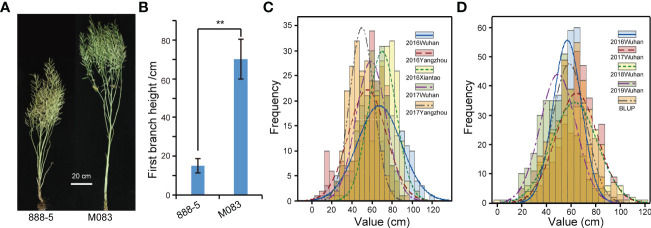
Parent phenotype and phenotypic frequency distribution of recombinant inbred lines (RILs) and natural accessions. **(A)** The mature stage phenotype of 888-5 and M083. Scale bar = 20 cm. **(B)** Comparison of first branch height (FBH) between 888-5 and M083. Data are presented as means ± SD (n ˃ 20). The double asterisks represent a significant difference determined *via* Student’s *t*-test at *P*< 0.01. **(C)** FBH phenotypic frequency distribution in RILs under five environments. **(D)** FBH phenotypic frequency distribution in 324 collected accessions under four consecutive years and best linear unbiased prediction (BLUP).

The FBH values of 888-5 and M083 was 14.9 ± 3.8 cm and 70.5 ± 10.2 cm, respectively, which showed highly significant difference in the field trial (**Figures 1A, B**). The phenotypic data of the FBH of RIL lines were investigated in five environments (**Supplementary Table 3**), and all displayed normal frequency distributions with continuous variation (**Figure 1C**). Significant variation among the FBH genotypes was observed: for instance, the minimum FBH was 6.15 cm, while the maximum was 121.4 cm, and the coefficient of variation ranged from 19.84 to 40.94% (**Table 1**), which showed great distinction. In the association population, the phenotypic frequency distributions of FBH in the four environments and BLUP all presented approximately continuous and normal distributions (**Figure 1D**; **Supplementary Table 5**), indicating that the group was suitable for association analysis. Significant variation was also observed in the association population, for example, FBH ranged from 23.41 to 99.11 cm, with an average of 58.75 cm in BLUP; the coefficient of variation was 23.07% (**Table 1**). The coefficient of variation was greater than 19.84% in both RIL and association populations (**Table 1**), which indicated dispersion measures were high among accessions or lines. Broad-sense heritability (*H* ^2^) of FBH in linkage and association populations were 88.96% and 92.61% with significant variation (*P*< 0.01), respectively (**Supplementary Table 4**).

**Table 1:** Phenotypic variation in the linkage and association populations.

Population	Env	Min (cm)	Max (cm)	Mean (cm)	SE	SD	Var	CV (%)	Kurtosis	Skewness
RIL	2016Wuhan	7.33	121.40	67.54	1.52	21.95	481.99	32.51	-0.225	-0.310
	2016Yangzhou	6.15	93.07	52.81	1.49	21.62	467.47	40.94	-0.637	-0.801
	2016Xiantao	27.38	95.87	70.41	0.96	13.97	195.13	19.84	-0.617	-0.093
	2017Wuhan	14.00	105.50	59.34	1.03	14.95	223.47	25.19	-0.267	0.449
	2017Yangzhou	19.41	78.50	50.13	0.83	12.08	145.83	24.09	0.099	-0.617
GWAS	2016Wuhan	24.96	91.19	57.35	0.64	11.59	134.23	20.20	-0.160	-0.007
	2017Wuhan	8.62	116.30	64.73	0.97	17.47	305.07	26.98	-0.471	0.703
	2018Wuhan	1.95	118.79	62.65	1.07	19.21	369.15	30.67	-0.192	0.467
	2019Wuhan	5.60	94.60	49.72	0.81	14.65	214.61	29.47	0.028	-0.133
	BLUP	23.41	99.11	58.75	0.75	13.55	183.65	23.07	-0.137	-0.089

Env, environment; Min, minimum value; Max, maximum value; Mean, mean value; SE, standard error; SD, standard deviation; Var, variance; CV, coefficient of variation.

### FBH association analysis

**Figure 2:**
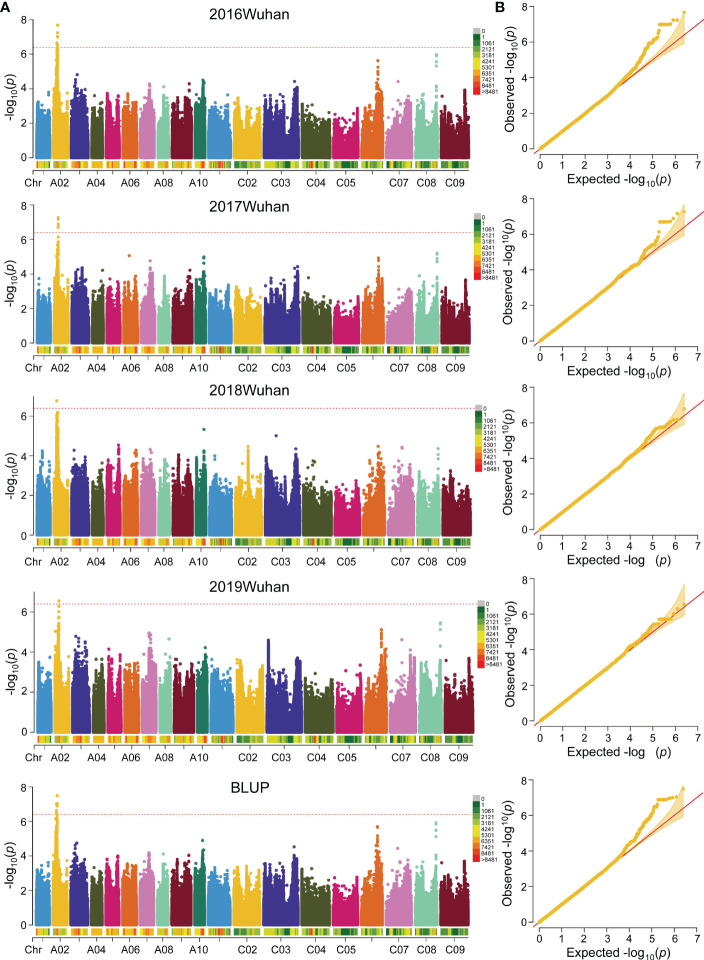
Genome-wide association study (GWAS) of FBH trait in 324 accessions. **(A)** Manhattan plot of FBH identified by GWAS in four consecutive years and BLUP based on a mixed linear model (MLM) of Efficient Mixed-Model Association eXpedited (EMMAX). The red dotted lines and color blocks represent threshold lines and SNP number in 1 Mb window size, respectively. **(B)** Quantile-Quantile plot of FBH in four consecutive years and BLUP.

Based on the whole genome sequencing (WGS) data, a total of 2,465,230 high-quality SNPs (minor allele frequency, MAF > 0.05, missing rate< 0.5) were filtered in previous studies and the LD distances of the A and C sub-genomes were 21 and 128 kb (r^2^ > 0.2), respectively. The quantile-quantile plots for GWAS based on the four environments and BLUP values of FBH implied that the associations were well controlled for population structure, and the Manhattan plots revealed only one significant peak located on ChrA02 in the four environments and BLUP (**Figures 2**). At a significance level of *P*< 1/2,465,230, 26 significant SNPs were mapped on A02 chromosome in BLUP within the interval of 6.92-7.11 Mb between SNPs p6926697 and p7113686. (**Figure 2A** **;** **Supplementary Table 6**). These results suggest the presence of an important genetic locus in A02 that regulates FBH.

### QTL linkage mapping of FBH

**Figure 3:**
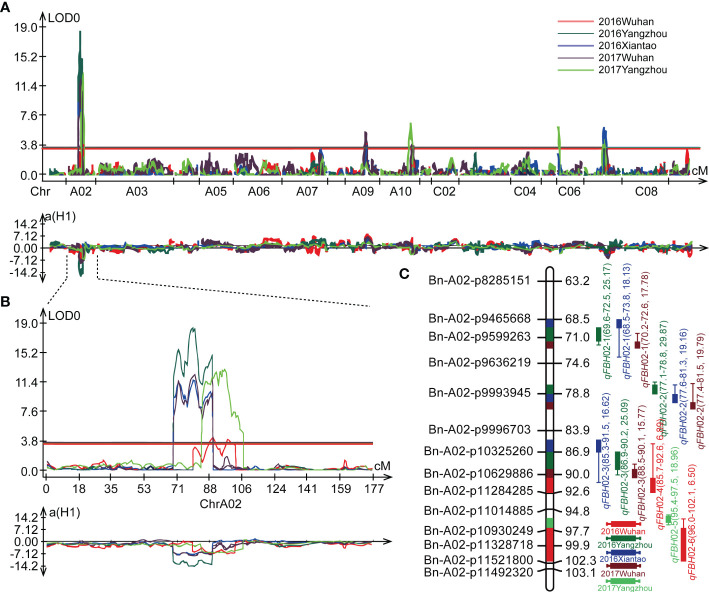
Quantitative trait loci (QTL) identification for FBH in five environments. **(A)** QTL distribution on the whole genome; diverse colors represent the QTL identified in different environments. **(B)** Localization of the identified QTLs on linkage group A02 in different environments. **(C)** Genetic markers and QTL localizations on linkage group A02.

Based on the modified *B.napus* 60 K SNP array and a high density genetic map developed in a previous study (Zhang et al., 2019b), a total of 19 QTLs were detected for FBH in single environment using WinQTL Cartographer 2.5 (**Figure3A**; **Table 2**). These QTLs were distributed on linkage groups A02, A09, A10, C06, and C07, with phenotypic variation explained (PVE) rates ranging from 4.87 to 29.87% (**Table 2**). Among these QTLs, six were located in an approximate region on linkage group A02, and *qFBH*02-1, *qFBH*02-2, and *qFBH*02-3 were repeatedly detected in more than one environment, with the PVE ranging from 15.77 to 29.87% (**Figures 3B, C**; **Table 2**). In addition, *qFBH*02-5 demonstrated a PVE as high as 18.96%, but it was only detected in E5. Simultaneously, four QTLs were detected in group C07 under all environments, except E1, and explained 5.61 ~ 9.02% of phenotypic variation (**Table 2**). Among the 19 QTLs, *qFBH*09-1, *qFBH*09-2 and *qFBH*09-3 low-FBH alleles came from 888-5, and the remaining high-FBH alleles QTLs were provided by M083.

**Table 2:** Putative QTLs for rapeseed FBH in RIL population under different environments.

QTL	Peak position (cM)	PVE (%)	Additive effect	LOD	Interval (cM)	Env
*qFBH*02-1	71.1	25.17	-12.94	14.67	69.6-72.5	E2
	71.1	18.13	-7.13	14.32	68.5-73.8	E3
	71.1	17.78	-7.57	14.45	70.2-72.6	E4
*qFBH*02-2	78.8	29.87	-14.16	8.79	78.2-78.8	E2
	78.8	19.16	-7.32	8.88	77.6-81.3	E3
	78.8	19.79	-8.10	8.94	77.4-81.5	E4
*qFBH*02-3	89.5	16.62	-7.14	11.43	85.3-91.5	E3
	89.5	25.09	-13.17	13.98	86.9-90.2	E2
	89.5	15.77	-7.33	11.29	88.5-90.1	E4
*qFBH*02-4	90.0	6.89	-6.96	4.69	85.7-92.6	E1
*qFBH*02-5	95.9	18.96	-6.32	5.38	95.4-97.5	E5
*qFBH*02-6	97.7	6.50	-6.83	5.12	96.0-102.1	E1
*qFBH*09-1	128.8	6.93	5.82	3.74	122.4-130.4	E1
	128.8	4.87	4.82	3.81	128.3-130.4	E2
	128.8	7.69	4.25	4.21	123.1-131.6	E4
*qFBH*09-2	134.2	5.07	4.90	2.76	132.8-140.8	E2
*qFBH*09-3	137.6	5.71	3.43	4.57	134.8-144.8	E3
*qFBH*10-1	205.5	8.65	-3.73	4.61	194.6-209.5	E5
*qFBH*10-2	211.7	8.64	-3.65	5.25	209.5-213.7	E5
*qFBH*10-3	212.7	6.09	-3.75	2.55	206.6-214.8	E4
*qFBH*10-4	218.5	5.98	-3.71	4.45	214.8-220.9	E4
*qFBH*16-1	2.0	8.20	-3.48	3.67	0.0-4.3	E5
*qFBH*16-2	8.7	6.56	-3.14	3.72	7.5-10.0	E5
*qFBH*17-1	127.0	6.61	-4.09	4.05	122.9-130.7	E4
	127.0	5.61	-2.97	4.12	122.9-129.1	E5
*qFBH*17-2	133.8	9.02	-4.45	3.73	130.3-135.4	E3
	134.2	5.86	-3.04	3.63	129.1-134.3	E5
*qFBH*17-3	145.6	6.84	-5.92	4.06	413.5-148.4	E2
	145.6	8.46	-4.32	3.89	145.2-149.2	E3
*qFBH*17-4	153.8	5.70	-5.45	3.67	151.2-157.1	E2

PVE, phenotypic variation explained; LOD, logarithm of odds; Env, environment; E1, 2016Wuhan; E2, 2016Yangzhou; E3, 2016Xiantao; E4, 2017Wuhan; E5, 2017Yangzhou.

### Overlaps between linkage and association mapping

**Figure 4:**
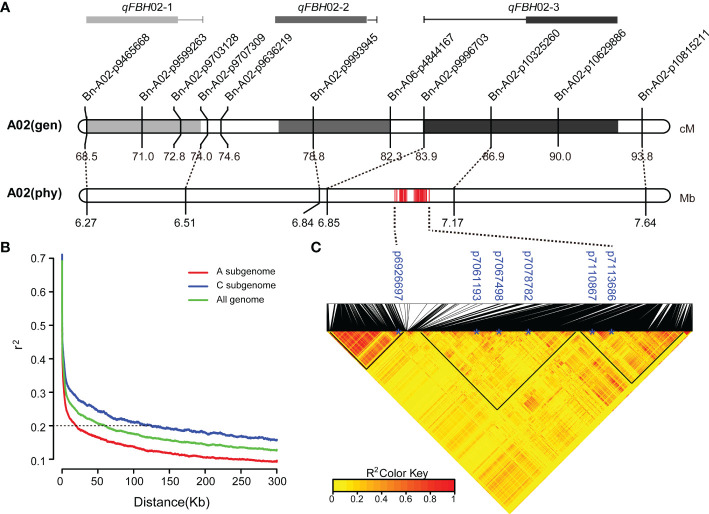
Co-localization of *qA02.FBH*. **(A)** The genetic location corresponds to the physical location. **(B)** Linkage disequilibrium (LD) decay of the **(A, C)** subgenomes and the whole genome. **(C)** LD heatmap surrounding the peak on A02. The black triangles represent closely linked loci.

Integrated linkage and association mapping results, showed that the 26 significant SNP loci in GWAS and *qFBH*02-3 obtained *via* linkage mapping had overlapping regions (**Figure 4A**), which indicated that the overlapped region could be considered the major QTL for FBH trait. LD decay analysis of 324 rapeseed germplasm resources showed that the decay distances (r^2^ > 0.2) of sub-genomes A and C were approximately 20 kb and 60 kb, respectively (**Figure 4B**). The attenuation distance of sub-genome A was significantly smaller than that of sub-genome C, indicating that sub-genome A had a higher degree of chromosome variation and genetic diversity, whereas sub-genome C was more stable. Simultaneously, LD heatmap analysis 60 Kb upstream and downstream of the significant SNPs revealed that three tightly linked blocks within an interval of 6.89 - 7.12 Mb on chromosome A02, named *qA02.FBH* (**Figure 4C**).

### Candidate gene predictions

**Figure 5:**
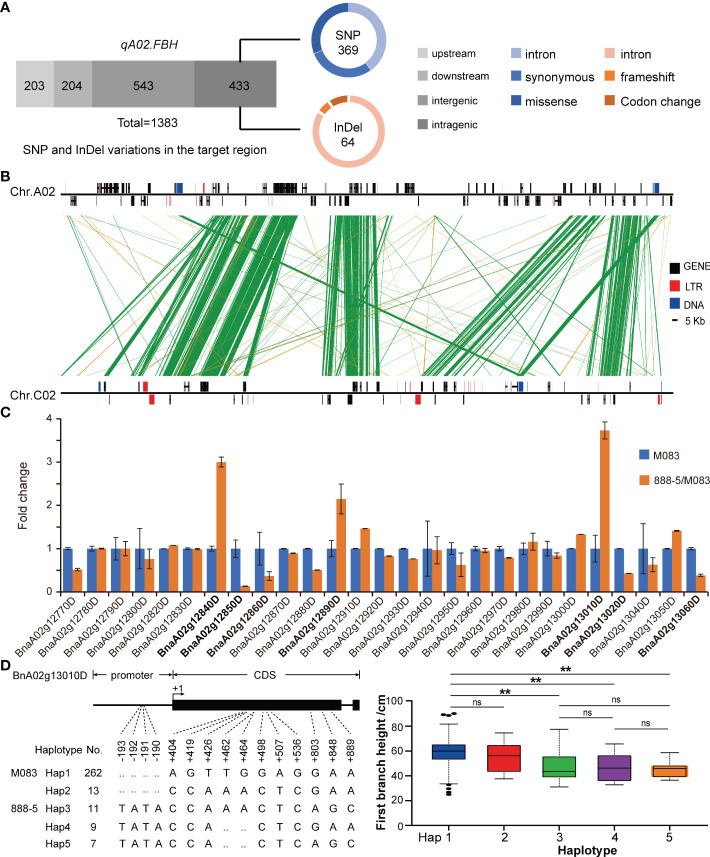
Candidate gene prediction *via* manifold analysis. **(A)** The identified variations in *qA02.FBH*. **(B)** Synteny analysis for *qA02.FBH* between subgenomes. **(C)** Expression levels of annotated genes in *qA02.FBH*. **(D)** Haplotype analysis of *BnaA02g13010D* based on association population in BLUP. Data are presented as means ± SD. The double asterisks represent a significant difference determined *via* Student’s *t*-test at *P* < 0.01. ns represents no significance.

The *qA02.FBH* region covers 230 kb and contains 31 annotated genes according to the reference genome annotation (Darmor-*bzh*), three of which (*BnaA02g12830D*, *BnaA02g12900D*, and *BnaA02g12930D*) with several hundreds of nucleotides had no orthologous genes in *Arabidopsis*. Four genes, *BnaA02g12770D*, *BnaA02g12780D*, *BnaA02g12910D*, and *BnaA02g13070D*, encoded unknown proteins. The remaining genes encoded functional proteins, including four pairs of genes (*BnaA02g12870D* and *BnaA02g12880D*, *BnaA02g12940D* and *BnaA02g12950D*, *BnaA02g12980D* and *BnaA02g12990D*, *BnaA02g13030D* and *BnaA02g13040D*) had the same functions as orthologous gene in *Arabidopsis* (**Supplementary Table 7**). At the DNA level, the SNP and InDel variations of the *qA02.FBH* between the two parents were analyzed using deep re-sequencing data (**Figure 5A**). In total, 1,398 variations were identified, most of which were located in the intergenic and intragenic region of the candidate genes, and 114 SNPs and 10 InDels resulting in missense mutations and frameshift or codon changes in the coding region, respectively (**Supplementary Tables 8**, **9**).

Synteny analysis revealed that, the syntenic block of *qA02.FBH* ranged from 12,397,768 to 13,133,808 bp on chromosome C02 (**Figure 5B**). There was no syntenic genes for *BnaA02g12830D*, *BnaA02g12840D*, *BnaA02g12890D*, *BnaA02g12910D*, *BnaA02g12920D*, and *BnaA02g12940D* in the syntenic block (**Table 3**). In contrast, there were no QTLs or significant SNPs identified in the syntenic block based on the association and linkage mapping results (**Figures 2A**, **3A**), suggesting that these six genes should be considered as candidate genes. Simultaneously, seven genes, *BnaA02g12840D*, *BnaA02g12850D*, *BnaA02g12860D*, *BnaA02g12890D*, *BnaA02g13010D*, *BnaA02g13020D*, and *BnaA02g13060D*, were differentially expressed between SAM tissues of the two parents, as demonstrated using RT-qPCR (**Figure 5C** **;** **Table 3**). The variations and haplotype results of above eleven genes revealed that *BnaA02g13010D* had TATA variation in the promoter region, resulting in a significant difference in BLUP (**Figure 5D** **;** **Table 3**). A BLAST search revealed that *BnaA02g13010D* was annotated as a transcription factor with a TCP (TB1, CYC, PCF) domain (*BnaA02.TCP1*) gene in BRAD (http://brassicadb.cn/), and its homologous genes were associated with plant architecture in *Arabidopsis* (Guo et al., 2010; Gao et al., 2015) and maize (Dixon et al., 2020).

**Table 3:** Candidate genes for FBH in the *qA02.FBH* interval.

*B*.*napus* gene ID	*Ath* homolog	Gene annotation	Variations	Syntenic gene	log_2_fold change
*BnaA02g12830D*	#N/A	#N/A	√	√	-0.014
*BnaA02g12840D*	*AT5G38430.1*	Ribulose bisphosphate carboxylase (small chain) family protein	√	√	1.586
*BnaA02g12850D*	*AT1G67100.1*	LOB domain-containing protein 40	×	×	-2.913
*BnaA02g12860D*	*AT1G67110.1*	cytochrome P450, family 735, subfamily A, polypeptide 2	√	×	-1.444
*BnaA02g12890D*	*AT5G40480.1*	embryo defective 3012 (EMB3012), embryo development ending in seed dormancy	√	√	1.103
*BnaA02g12910D*	*AT1G67170.1*	unknown protein	√	√	0.550
*BnaA02g12920D*	*AT1G67180.1*	zinc finger (C3HC4-type RING finger) family protein/BRCT domain-containing protein	√	√	-0.271
*BnaA02g12940D*	*AT2G31470.1*	F-box and associated interaction domains-containing protein	√	√	0.312
*BnaA02g13010D*	*AT1G67260.2*	TCP family transcription factor	√	×	1.900
*BnaA02g13020D*	*AT1G67780.1*	Zinc-finger domain of monoamine-oxidase A repressor R1 protein	√	×	-1.221
*BnaA02g13060D*	*AT1G67320.2*	DNA primase, large subunit family, DNA replication, synthesis of RNA primer	√	×	-1.381

### *BnaA02.TCP* altered plant architecture in transgenic *Arabidopsis*

**Figure 6:**
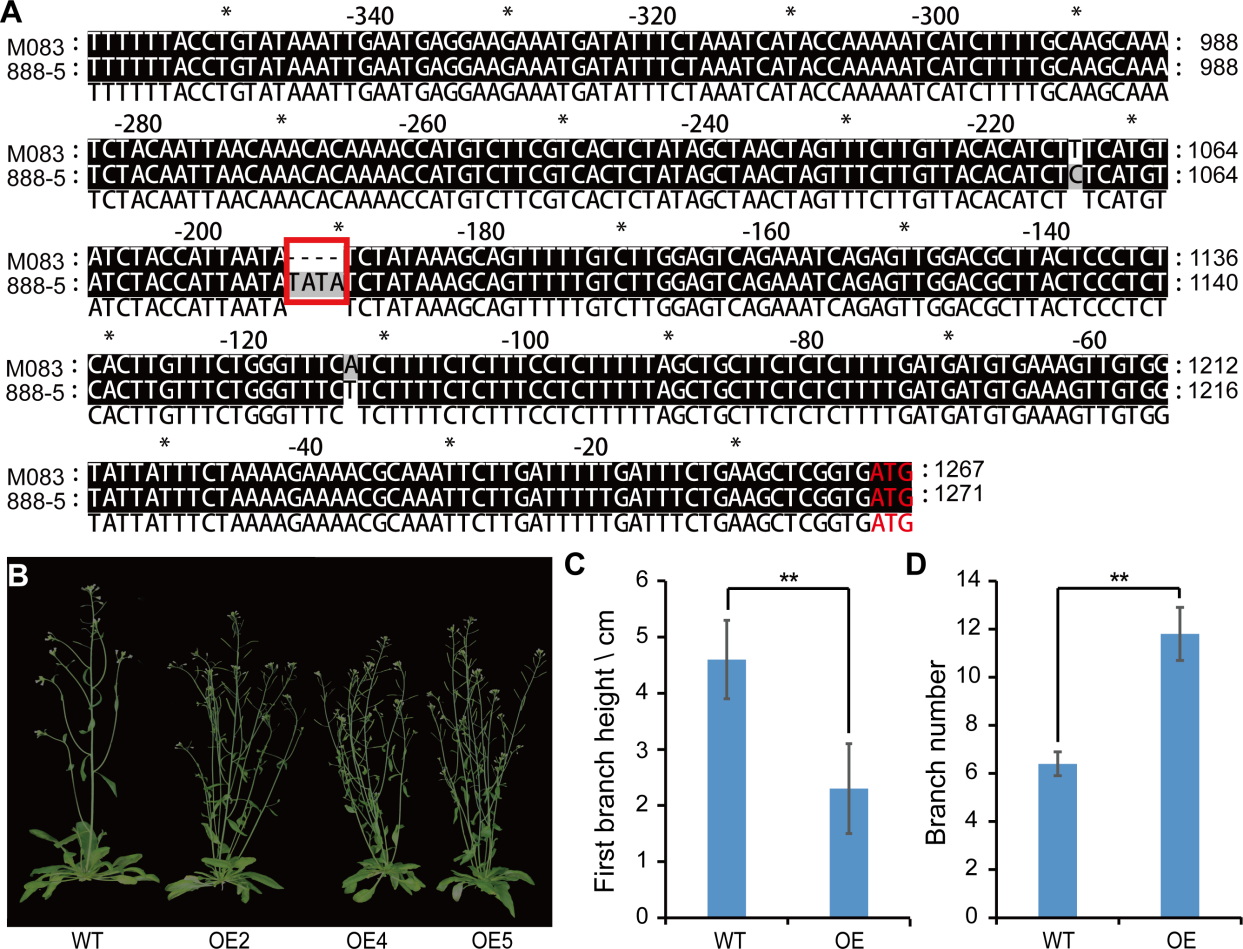
Sequence analysis and phenotypic comparison of wild type (WT) plants and the *BnaA02.TCP1* ^M083^-OE lines in *Arabidopsis*. **(A)** Alignment of *BnaA02.TCP1* promotor sequences between 888-5 and M083. The red rectangle represents the nucleotide change between 888-5 and M083. **(B)** Phenotype comparison of WT and *BnaA02.TCP1* ^M083^ overexpression (OE) lines in *Arabidopsis* at the grain filling stage. **(C, D)** Digital comparison of **(C)** FBH and **(D)** branch number between WT and OE lines in *Arabidopsis*. Data are presented as means ± SD (n = 10). The double asterisks represent a significant difference determined using Student’s *t*-test at *P* < 0.01.

Sequencing results showed that *BnaA02.TCP1* ^M083^ had a TATA deletion in the promoter region and 14 missense mutations in the coding region compared with *BnaA02.TCP1* ^888-5^ (**Figure 6A** **;** **Supplementary Figure 1**).*BnaA02.TCP1* expression was higher in 888-5 SAM than in M083 SAM (**Figure 5C**). Consequently, an overexpression (OE) vector containing *BnaA02.TCP1* ^M083^ was transferred into wild-type *Arabidopsis* (col-0). Transgenic lines were validated by qRT-PCR and three highly expressed lines were randomly selected for phenotyping (**Supplementary Figure 2** **)**. At the mature stage, *BnaA02.TCP1* ^M083^-OE transgenic plants exhibited higher FBH and more branches than the wild type plants (**Figures 6B-D**). Phylogenetic tree analysis of *BnaA02.TCP1* showed that *AtTCP1* was most similar in sequence, suggesting that they are homologous and perform the same function. These findings implied that *BnaA02.TCP1* may respond to rapeseed plant architecture development, as in *Arabidopsis*.

## Discussion

### Comparisons with previous study of FBH

Improvements in traits of agronomic importance are the primary objective of crop improvement programs. Most agronomic traits are controlled by complex or quantitative loci (Abe et al., 2012). With the increase in rapeseed yield demand, labor cost, and decrease of farmer profits, many challenges require a quantum leap in seed yield, reducing yield loss caused by biotic and abiotic stresses, and applying gene editing and genome breeding biotechnology tools (Liu et al., 2022). As a component of plant architecture, FBH is not as important as other traits, such as plant height and flowering time, but it is also a concern for breeders. Appropriate branch height can significantly increase rapeseed yield during practical production (Cai et al., 2016), so the underlying molecular mechanisms must be elucidated. However, only a few studies have reported on FBH in rapeseed, and studied on cloning genes related to FBH still lacking.

Chen et al. (2007) identified 10 QTLs distributed on chromosomes A02, A07, A08, C04, C06, and C07, associated with the height of the lowest primary effective branch using two rapeseed populations. Another study detected four peak SNPs located on chromosomes A02, A07, A08, and A09 using GWAS with 333 collected rapeseed accessions (Zheng et al., 2017). Candidate genes for FBH were identified, but no functional genes discovered or demonstrated. In this study, we used an F10 RIL population derived from M083 × 888-5 cross in QTL analysis of FBH. Nineteen QTLs were identified on chromosomes A02, A09, A10, C06, and C07 (**Figure 3A**; **Table 2**). The QTLs located on chromosome A10 were different from QTLs associated with FBH in previous studies, while the QTLs on chromosome A02 were consistent with previous study, which suggests that our results were reliable, novel, and worthy of further investigation. Using the same RIL population, Zhang et al. (2019b) performed QTL mapping for flowering time and *Sclerotinia* resistance traits. Nineteen QTLs for flowering time and twenty-five QTLs for *Sclerotinia* resistance were identified. Interestingly, *qFBH*02-1 and *qFBH*02-3 identified in the current study co-localized with QTLs controlling flowering time. *qFBH*02-3 and *qFBH*02-4 were consistently correlated with the QTLs of *Sclerotinia* resistance, indicating that *qFBH*02-3 might have pleiotropic effects on controlling FBH, flowering time, and *Sclerotinia* resistance.

### Association and linkage mapping combination for rapid candidate interval identification

QTL identification, followed by mapping and cloning of candidate genes/QTLs are central to agronomic traits analysis. Linkage mapping, a traditional QTL mapping method, was used to accurately and slowly detect QTL regions with genetic makers. GWAS has been widely used to detect variations associated with complex agronomic traits in natural populations with the development of whole-genome sequencing over last 10 years, which may circumvent the cumbersome procedures for fine mapping (Jaganathan et al., 2020; Wang et al., 2020a). The complex genetic background of allotriploid rapeseed and the agronomic traits usually affected by the environment complicate integral and accurate detection using a single method. Therefore, integrating association and linkage mapping would be quick and accurate. Previous studies have used this approach to map QTL in crops (Sun et al., 2016; Han et al., 2018; Wang et al., 2020b; Zhang et al., 2020). In this study, only a single locus contained 26 SNPs associated with the FBH trait was identified in the natural population with GWAS (**Figure 2A**), which is very different from linkage mapping results (**Table 2**). The reasons may be the association population size was small, SNPs with low MAF were filleted out (MAF< 0.05), and trait was influenced by environment (Qiu et al., 2015). Therefore, major QTL could be detected but minor QTLs can’t in small panel size (Si et al., 2016). Meanwhile, these significant SNPs were co-located with *qFBH*2-3, detected using linkage mapping in multiple environments and exhibited high phenotypic variation (**Figure 4**). Overall, the findings of the present study suggest that the *qA02.FBH* interval would be the major QTL for FBH (**Figure 4C**).

### *BnaA02.TCP* possibly affected rapeseed plant architecture by mediating strigolactone signaling

Hormones are crucial regulators of plant growth and development. Several studies have demonstrated that strigolactone is an important factor that regulates axillary bud growth and a determinant of plant architecture (Doebley et al., 1995; Martín-Trillo and Cubas, 2010; Dixon et al., 2020; Wang et al., 2020c). In plants, it remains unclear which and how genes involved in the strigolactone pathway could be utilized in breeding, but one possibility is that strigolactones regulate transcription of the TCP-domain transcription factor in shoot branching (Dun et al., 2012). In this study, *BnaA02.TCP1* was differentially expressed at the transcriptional level in parental SAM tissues owing to TATA variation in the promoter region (**Figures 5D**, **6A**). *BnaA02.TCP1* ^M083^-OE plants showed lower FBH and more branches compared with wild type in *Arabidopsis* (**Figures 6B-D**). Although *BnaA02.TCP1* over-expression and loss-of-function in rapeseed and the proteins interactions remain elusive in this study, the above findings are still credible, because rapeseed originates from *Arabidopsis* (Chalhoub et al., 2014). Therefore, we concluded that BnaA02.TCP1 closely related *Arabidopsis* TCP proteins, may be involved in strigolactone pathway activity to regulate the growth of rapeseed axillary buds.

## Conclusion

In this context, the genetic mechanism underlying FBH was analyzed through multiple strategies (integrating GWAS, linkage mapping, and gene expression analysis) in *B. napus*. In summary, 26 SNPs and 19 QTLs associated with FBH were identified in 324 accessions and RIL population derived from M083 and 888-5, respectively. Meta-analysis revealed that one unique QTL for FBH was co-located on chromosome A02. An integrated analysis of allelic variations, syntenic genes, and DEGs identified 11 candidate genes for FBH. Among 11 genes, *BnaA02g13010D* was confirmed as the target gene according to haplotype analysis and full-length gene sequencing, which revealed that TATA insertion/deletion in the promoter region was closely linked to significantly phenotypic differences. *BnaA02.TCP1* ^M083^ overexpression resulted in decreased branch height and increased branch number in *Arabidopsis*. Therefore, our data suggested that *BnaA02.TCP1* might play important role as regulator of FBH and provided a genetic basis for shaping ideal architecture in *B. napus*.

## Data availability statement

The original contributions presented in the study are publicly available. This data can be found here: NGDC (https://ngdc.cncb.ac.cn/gsa), CRA008925.

## Author contributions

ZD, ZL, YX, JH, XCh, and SL designed the research. ZD, CZ, XCu, YL, MT, CT, and JH performed the experiments. ZD and JH analyzed the data. YL provided the materials and phenotypic data. ZD, XCh, and JH wrote and revised the manuscript. All authors contributed to the article and approved the submitted version.

## Supplementary material

Supplementary Figure 1 Alignment of the deduced amino acid sequence between 888-5 and M083. The red rectangle represents the TCP domain.

Supplementary Figure 2 Expression levels of *BnaA02.TCP1* in wild-type (WT) and transgenic *Arabidopsis* plants (OE2, OE4, and OE5).
