# Supplementary figures and images for: Integrating GWAS, linkage mapping and gene expression analyses reveal the genetic control of first branch height in Brassica napus L
Source: Front Plant Sci. 2022 Dec 15;13:1080999. doi: 10.3389/fpls.2022.1080999 (PMC9798901; doi:10.3389/fpls.2022.1080999)

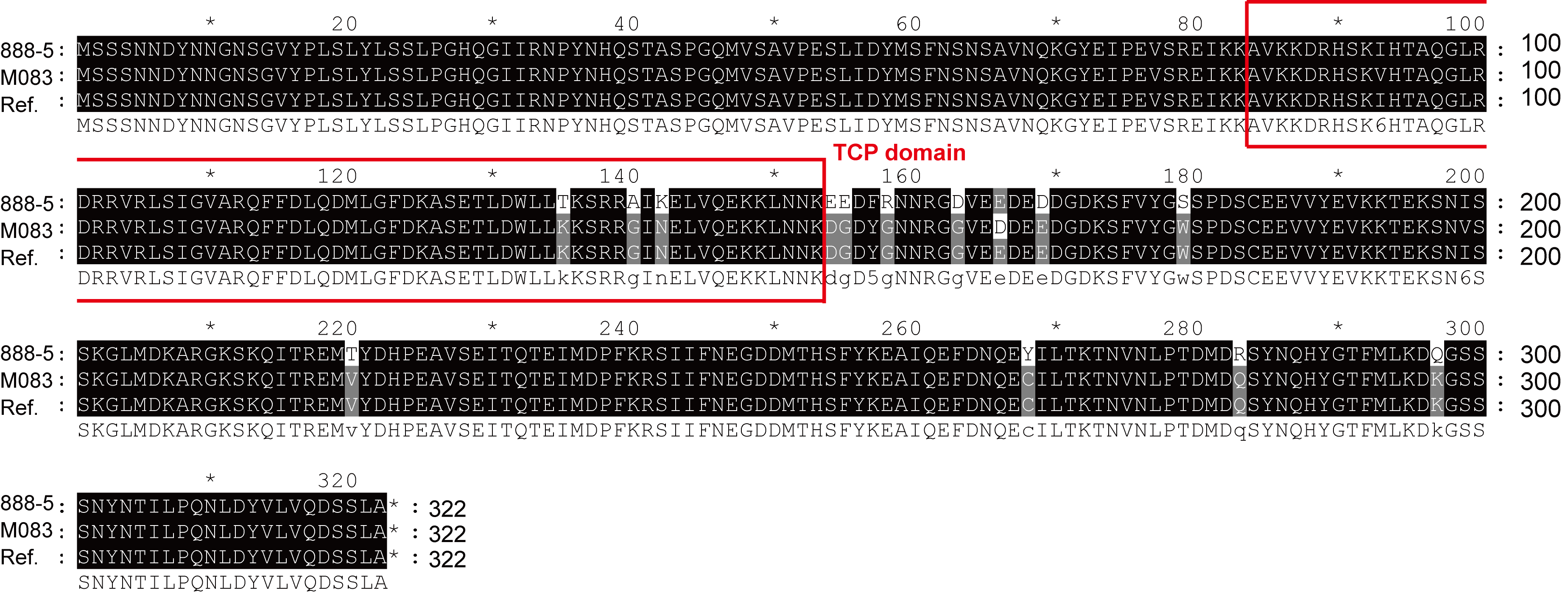

Supplement: Supplementary Figure 1 — Alignment of the deduced amino acid sequence between 888-5 and M083. The red rectangle represents the TCP domain. [file Image_1.tif]

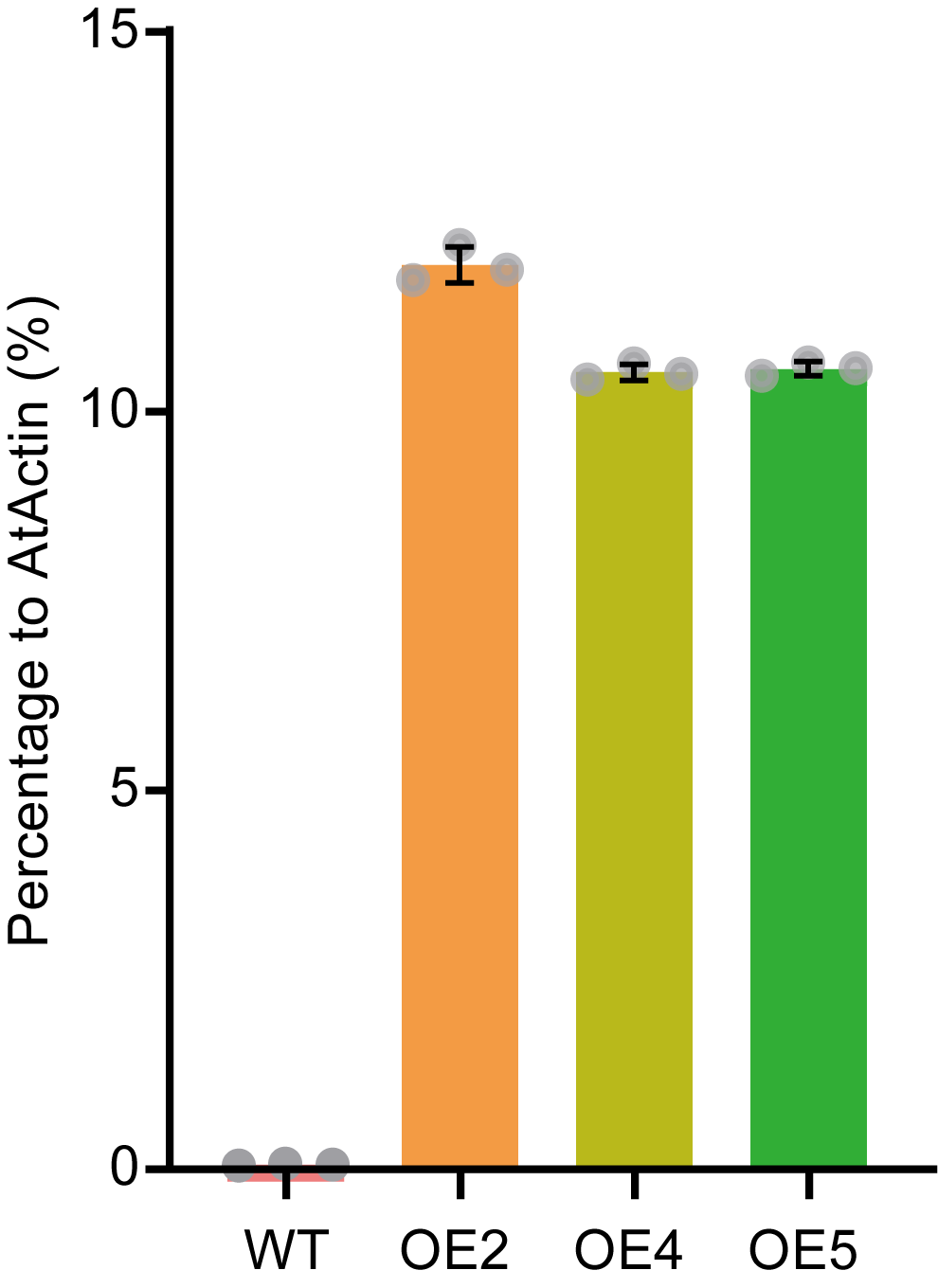

Supplement: Supplementary Figure 2 — Expression levels of BnaA02.TCP1 in wild-type (WT) and transgenic Arabidopsis plants (OE2, OE4, and OE5). [file Image_2.tif]
